# Mapping the research landscape of traditional Chinese medicine in insomnia management: a bibliometric study (2005–2024)

**DOI:** 10.3389/fneur.2025.1614948

**Published:** 2025-08-26

**Authors:** Chi Zhang, Xiaojie Yang, Jin Ye, Yuanxun Cai, Hanxiao Zhang, Yuelong Fang, Liying Zhang, Shuhe Cai

**Affiliations:** ^1^Fujian University of Traditional Chinese Medicine Subsidiary Rehabilitation Hospital, Fuzhou, China; ^2^Fujian Key Laboratory of Rehabilitation Technology, Fuzhou, China; ^3^Fujian University of Traditional Chinese Medicine, Fuzhou, China; ^4^Longyan Hospital of Traditional Chinese Medicine, Longyan, China

**Keywords:** insomnia, traditional Chinese medicine, bibliometric study, CiteSpace, VOSviewer, Bibliometrix

## Abstract

**Objective:**

Traditional Chinese Medicine (TCM) has shown unique benefits in insomnia management, but existing bibliometric studies on TCM for insomnia remain fragmented. This study, through bibliometric methods, systematically maps the research landscape of TCM in insomnia management from 2005 to 2024, with the objective to explore research hotspots and developmental trends, thereby providing references for future studies.

**Methods:**

This study retrieved English-language literature on the treatment of insomnia with Traditional Chinese Medicine from 2005 to 2024 in the Science Core Collection. The included literature was imported into CiteSpace, VOSviewer, and Bibliometrix R software packages to analyze annual publications, authors, countries/regions, institutions, journals, cited reference, and keywords, in order to explore the hotspots and trends in TCM treatment of insomnia.

**Results:**

A total of 738 articles were included. The number of annual publications in this field increased rapidly from 2016 to 2021. China was the country with the highest number of publications, among which Shanghai University of Traditional Chinese Medicine, Beijing University of Chinese Medicine, and Guangzhou University of Chinese Medicine were the top three institutions in terms of publication volume. Yeung, Wing-fai from Hong Kong Polytechnic University was the author with the highest number of publications. *Medicine* published 74 articles, the highest number among all journals. *Journal of Ethnopharmacology* and *Sleep* were, respectively, the most frequently cited and co-cited journals. The main keywords included sleep, randomized controlled trial, traditional Chinese medicine, acupuncture, etc. The research hotspots mainly focused on acupuncture, chemical components, cancer-related insomnia, and depression-related insomnia. The research focus is transitioning from clinical efficacy to mechanism research, especially the study of the chemical components of traditional Chinese medicine through network pharmacology may be a future research trend. Moreover, this field is paying more attention to insomnia subtypes such as comorbid insomnia and secondary insomnia.

**Conclusion:**

TCM treatment of insomnia is receiving increasing attention. It shows unique advantages in reducing drug dependence and managing comorbid insomnia. In the future, the evidence-based development of TCM should be promoted through mechanism research, multidisciplinary collaboration, stratified intervention, and the transformation of high-impact evidence, providing an integrated solution for global insomnia management.

## 1 Introduction

Insomnia is clinically defined as a sleep continuity disorder characterized by abnormalities in sleep latency, number of nocturnal awakenings, duration of awakenings during sleep maintenance, total sleep time, and sleep efficiency, accompanied by a cluster of symptoms that directly represent daytime functional impairment, such as somnolence, fatigue, physical discomfort (e.g., headache or generalized body aches), emotional disturbances, decline in cognitive function or occupational capacity, and concerns/discontent regarding sleep quality (1). Epidemiological studies indicate that approximately 10% of the adult population suffer from insomnia disorder, while around 20% experience occasional insomnia symptoms (2). Chronic insomnia not only causes cognitive decline (3) and immunosuppression (4), but also serves as a significant risk factor for cardiovascular diseases (5), metabolic syndrome (6), and mental disorders (7), emerging as a critical public health issue requiring urgent attention.

Current insomnia management is primarily divided into pharmacological and non-pharmacological interventions. Pharmacotherapy remains a common intervention, however, the 2017 guidelines from the American Academy of Sleep Medicine (AASM) emphasize that most hypnotic agents are supported by low-quality evidence and receive weak recommendations (8). Conventional medications, including benzodiazepines and non-benzodiazepine receptor agonists, are associated with side effects such as cognitive impairment, residual sedation, dependency risks, and high relapse rates upon discontinuation, thereby limiting their use to the short term (≤4 weeks) (9, 10). Although newer agents such as lemborexant and eszopiclone offer improved efficacy, concerns remain regarding their long-term safety and tolerability. Similarly, melatonin receptor agonists and orexin receptor antagonists present favorable safety profiles but currently lack robust clinical validation (11, 12).

Non-pharmacological interventions, particularly cognitive behavioral therapy for insomnia (CBT-I), are increasingly advocated as first-line treatments (13). The 2016 American College of Physicians (ACP) guideline on *“Management of Chronic Insomnia Disorder in Adults”* strongly advises CBT-I for all adult patients with chronic insomnia (14). Compared to pharmacotherapy, CBT-I demonstrates advantages in maintaining long-term efficacy, preserving daytime function, and reducing adverse event rates (15). Notwithstanding these benefits, initial CBT-I implementation may precipitate daytime fatigue, somnolence, emotional dysregulation, and cognitive difficulties (13). Nonetheless, limitations such as limited accessibility, high cost, delayed onset of efficacy, and potential contraindications in individuals with comorbid neuropsychiatric conditions (e.g., bipolar disorder, epilepsy, parasomnias) may restrict its clinical applicability (16).

In recent years, Traditional Chinese Medicine (TCM), as a vital component of complementary and alternative medicine (CAM), has attracted growing scientific attention for its multi-target therapeutic mechanisms, favorable safety profile, and holistic regulatory capacity. With a therapeutic history spanning thousands of years, TCM has documented insomnia management strategies since the *Huangdi Neijing (Yellow Emperor’s Inner Canon)*, which first described Banxia Decoction for insomnia treatment. Based on TCM theories, insomnia pathogenesis is closely linked to “yin-yang imbalance” and “visceral dysfunction.” In this study, TCM specifically refers to the therapeutic modalities guided by TCM theories, including internal interventions such as compound herbal formulas [e.g., Suanzaoren Decoction (17), Tianwang Buxin Dan (18)] and Chinese patent medicines (19), alongside external interventions such as acupuncture [e.g., electroacupuncture (20), intradermal acupuncture (21), auricular acupuncture (22), catgut embedding (22), moxibustion (23)], tuina (24), and qigong (25), demonstrating unique advantages in insomnia management.

With the rapid growth of TCM-related publications in recent years, there is a need to systematically evaluate the development and emerging trends in this field. Bibliometric analysis—a quantitative method for mapping scientific literature—has become an effective tool for assessing research hotspots, intellectual structures, and collaboration networks across disciplines. By analyzing metadata such as authorship, institutional affiliations, keywords, and citation patterns, bibliometrics provides a macroscopic view of knowledge production and evolution (26). Given that the past 20 years have witnessed the rapid internationalization of TCM, and considering the timeliness of research trends, along with the characteristics of the number of publication counts and citation counts on TCM treatments for insomnia before 2005, a concentrated analysis of recent literature is crucial for obtaining the latest progress. Although several bibliometric studies have explored specific aspects of TCM for insomnia, such as acupuncture (27, 28), nursing-based interventions (29), or treatments targeting yin-deficiency syndromes (30), these analyses remain fragmented. To date, no comprehensive bibliometric analysis has integrated both internal and external TCM interventions for insomnia within a unified framework.

Against the backdrop of TCM’s rapid internationalization and the fragmented status of existing bibliometric research on TCM for insomnia, this study addresses this research void by systematically conducting bibliometric and visual analytics on TCM for insomnia treatment spanning 2005–2024, constructing a holistic perspective on the current academic landscape. The primary objective is to explore research hotspots and developmental trends in the field. Ultimately, these findings will furnish theoretical foundations for future inquiries.

## 2 Materials and methods

### 2.1 Literature sources and data retrieval strategies

All data in this study were retrieved from the Web of Science Core Collection (WoSCC) on February 3, 2025. The search strategy was systematically designed as follows:

#1:((TI = (“Traditional Chinese medicine” OR TCM OR “Drugs, Chinese Herbal” OR “Acupuncture” OR “Acupuncture Therapy” OR “Acupuncture Treatment” OR “Acupuncture Treatments” OR “electroacupuncture” OR “Tuina” OR “Auriculotherapy” OR “Auriculotherapies” OR “moxibustion” OR “qigong”)) OR AB = (“Traditional Chinese medicine” OR “TCM” OR “Drugs, Chinese Herbal” OR “Acupuncture” OR “Acupuncture Therapy” OR “Acupuncture Treatment” OR “Acupuncture Treatments” OR “electroacupuncture” OR “Tuina” OR “Auriculotherapy” OR “Auriculotherapies” OR “moxibustion” OR “qigong”)) OR AK = (“Traditional Chinese medicine” OR “TCM” OR “Drugs, Chinese Herbal” OR “Acupuncture” OR “Acupuncture Therapy” OR “Acupuncture Treatment” OR “Acupuncture Treatments” OR “electroacupuncture” OR “Tuina” OR “Auriculotherapy” OR “Auriculotherapies” OR “moxibustion” OR “qigong”)
#2:((TI = (“Sleep Initiation and Maintenance Disorders” OR “DIMS” OR “Disorders of Initiating and Maintaining Sleep” OR “Sleeplessness” OR “Insomnia Disorder” OR “Insomnia”)) OR AB = (“Sleep Initiation and Maintenance Disorders” OR “DIMS” OR “Disorders of Initiating and Maintaining Sleep” OR “Sleeplessness” OR “Insomnia Disorder” OR “Insomnia”)) OR AK = (“Sleep Initiation and Maintenance Disorders” OR “DIMS” OR “Disorders of Initiating and Maintaining Sleep” OR “Sleeplessness” OR “Insomnia Disorder” OR “Insomnia”)
#3:#1 AND #2

**Figure 1:**
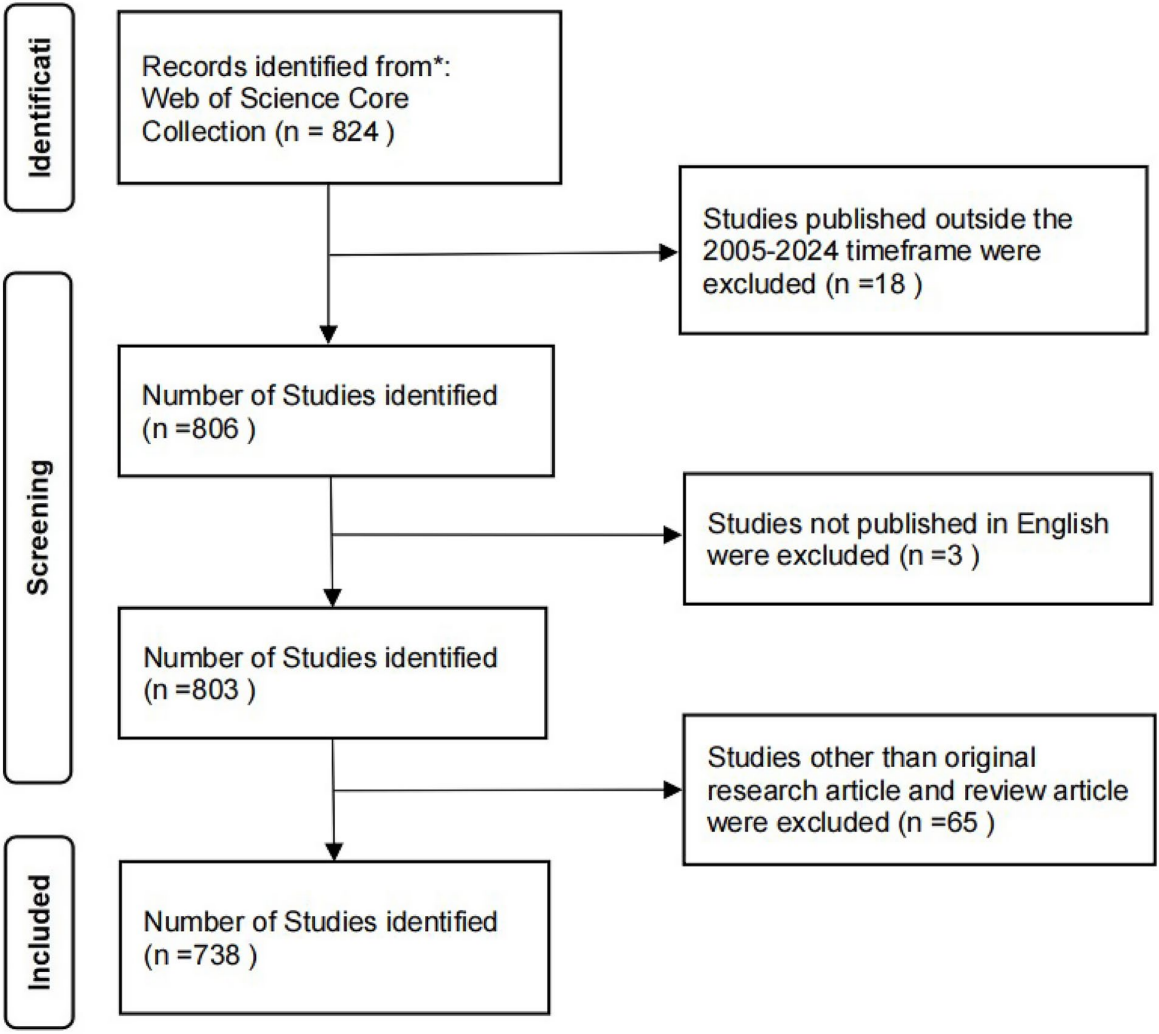
Selection process diagram.

The search parameters were constrained to English-language articles published between January 1, 2005, and December 31, 2024. Only “Article” and “Review Article” publication types were included. To ensure methodological rigor, two independent researchers performed the screening and evaluation processes. Any discrepancies in article selection were resolved through consultation with a third researcher. Following this protocol, 738 publications met the predefined inclusion criteria for bibliometric analysis. The retrieval flow chart is shown in Figure 1.

### 2.2 Data analysis

The raw datasets containing Full Record and Cited References were exported as Plain Text Files from the specified databases. Temporal evolution patterns of publication volume and citation counts were charted using Microsoft Excel 2016 (Microsoft Corp., Redmond, WA, United States). CiteSpace (31) (v6.3. R1) was deployed for institutional analysis, cited reference, and keyword mapping with the following configurations: Time Slicing spanning January 2005 to December 2024 (Years Per Slice = 1), comprehensive Term Source selection, and Selection Criteria set to g-index (k = 25). VOSviewer (32) (v1.6.20) was specifically employed for co-authorship networks (threshold: Minimum number of documents of a source = 5) and journal analysis, with Minimum number of documents of a source = 4 for cited journals and Minimum number of citations of a source = 20 for co-cited journals. Bibliometrix R package (33) (v4.4.2) was utilized for country/region profiling, journal evaluation, and keyword analysis, applying Filters to constrain Publication Year between 2005 and 2024.

Node dimensions reflect publication output in author/institutional networks, citation frequencies in journal/reference networks, and keyword occurrence counts; links represent collaborative relationships (author/institution), co-occurrence/co-citation linkages (journal/reference), or thematic associations (keywords), with links thickness scaled proportionally to connection intensity.

In CiteSpace clustering analysis, Modularity Q values exceeding 0.3 signify statistically significant cluster structures, while Weighted Mean Silhouette S scores above 0.5 denote conceptually reasonable groupings, with S > 0.7 indicating highly credible and efficient cluster formations. Furthermore, nodes attaining betweenness centrality greater than 0.1 in CiteSpace networks are recognized as possessing prominent mediating roles within the knowledge graph architecture, functioning as critical hubs that bridge distinct research domains or academic communities through strategic knowledge intermediation.

## 3 Results

### 3.1 Analysis of annual publications

**Figure 2:**
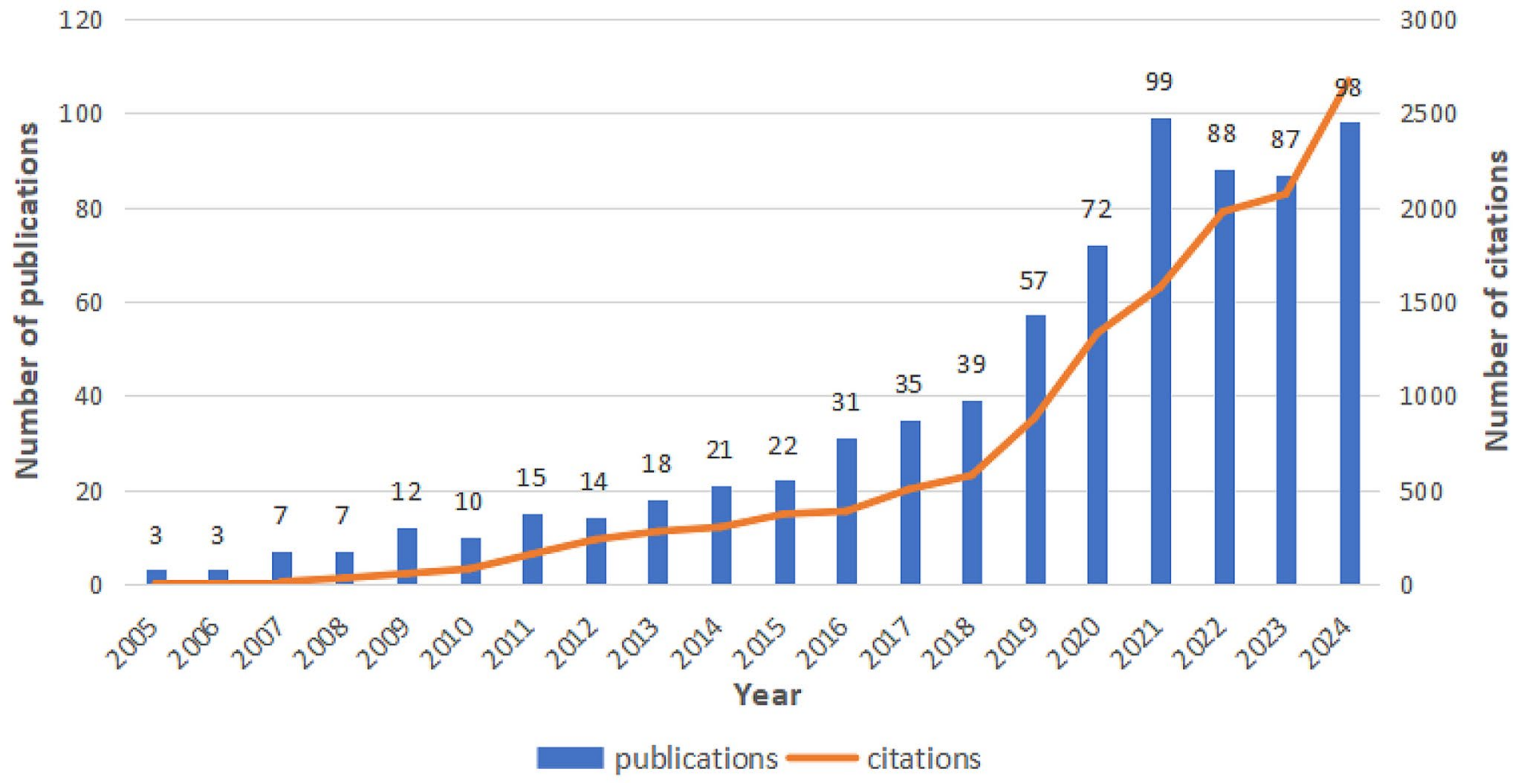
Trends in annual publication outputs of TCM in insomnia management.

Based on the Web of Science Core Collection database, this study conducted a statistical analysis of the number of publications on the application of traditional Chinese medicine therapies in the field of insomnia from 2005 to 2024. As shown in Figure 2, a total of 738 publications were identified by 2024, including 534 articles (72.36%) and 204 reviews (27.64%). From a temporal perspective, the annual publication output during 2005–2015 was in a phase of gradual accumulation, with fewer than 30 publications per year on average. A significant acceleration in publication growth was observed from 2016 to 2021, culminating in a peak of 99 publications in 2021, marking the most productive year in the past two decades. Although a slight decline in publication output occurred from 2022 to 2024 compared to the peak year, the numbers remained relatively high, with 98 publications recorded in 2024.

According to the Web of Science database, the 738 publications included in this study have been cited a total of 13,629 times, with an average citation count of 18.47 per publication. Analysis of citation trends reveals a steady increase in citations from 2005 to 2018, followed by a rapid growth phase from 2018 to 2024. The consistent growth in annual publication output and citation counts highlights that the application of traditional Chinese medicine therapies in the field of insomnia is an increasingly prominent and actively researched area.

### 3.2 Author/co-cited author analysis

**Figure 3:**
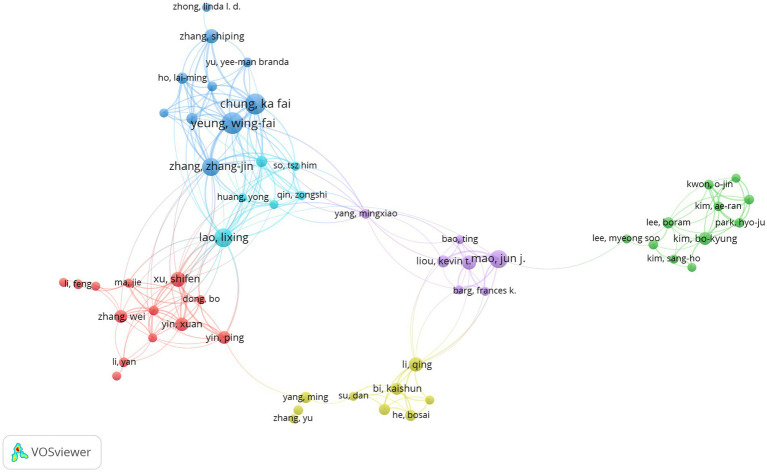
Network diagram of collaboration among authors in TCM for insomnia management.

Tables 1, 2 present the top 10 authors by publication volume and co-citation counts, respectively. Yeung, Wing-Fai from Hong Kong Polytechnic University emerged as the most prolific contributor with 27 publications in this field. With the top 10 authors contributing 22.9% of total outputs, indicating research concentration. Buysse, DJ from the University of Pittsburgh ranked as the most co-cited author (229 citations). A recipient of the Highly Cited Researcher Award, Buysse served as a key developer of the well-known Pittsburgh Sleep Quality Index (PSQI) in 1993, which has become a widely adopted clinical assessment tool. Notably, Yeung, Wing-Fai not only leading in publication volume, but also ranked third in co-citation counts (222 citations), underscoring his expertise in this domain. The collaborative network map generated using VOSviewer (Figure 3) visualizes author partnerships, where node size corresponds to publication volume and connection thickness reflects collaboration strength. The analysis reveals strong collaborative ties among Yeung, Wing-Fai, Chung, Ka-Fai, and Zhang, Zhang-Jin, forming a prominent research cluster in this field.

**Table 1:** The top 10 authors by publication volume.

Rank	Author	Institution	Counts
1	Yeung, Wing-Fai	Hong Kong Polytechnic University	27
2	Chung, Ka Fai	University of Hong Kong	25
3	Lao, Lixing	National Central University	20
4	Zhang, Zhang-Jin	University of Hong Kong	18
5	Mao, Jun J.	Memorial Sloan Kettering Cancer Center	18
6	Xu, Shifen	Shanghai University of Traditional Chinese Medicine	13
7	Wu, Wenzhong	Heilongjiang Red Cross Sengong Gen Hosp Harbin Medical University	12
8	Zhang, Shiping	Harbin Institute of Technology	12
9	Li, Qing	Memorial Sloan Kettering Cancer Center	12
10	Garland, Sheila N.	Beatrice Hunter Canc Res Inst	12

**Table 2:** The top 10 authors by co-citation counts.

Rank	Co-cited author	Institution	Citations
1	Buysse, DJ	University of Pittsburgh	229
2	Morin, CM	Laval University	225
3	Yeung, WF	Hong Kong Polytechnic University	222
4	Riemann,D	University of Freiburg	158
5	Chung, KF	University of Hong Kong	122
6	Yin, X	Shanghai University of Traditional Chinese Medicine	101
7	Garland, Sheila N.	Beatrice Hunter Canc Res Inst	99
8	Mao, JJ	Memorial Sloan Kettering Cancer Center	92
9	Ohayon, MM	Stanford University	85
10	Bastien, CH	Laval University	83

### 3.3 Analysis of country/regional and institutional collaboration networks

**Figure 4:**
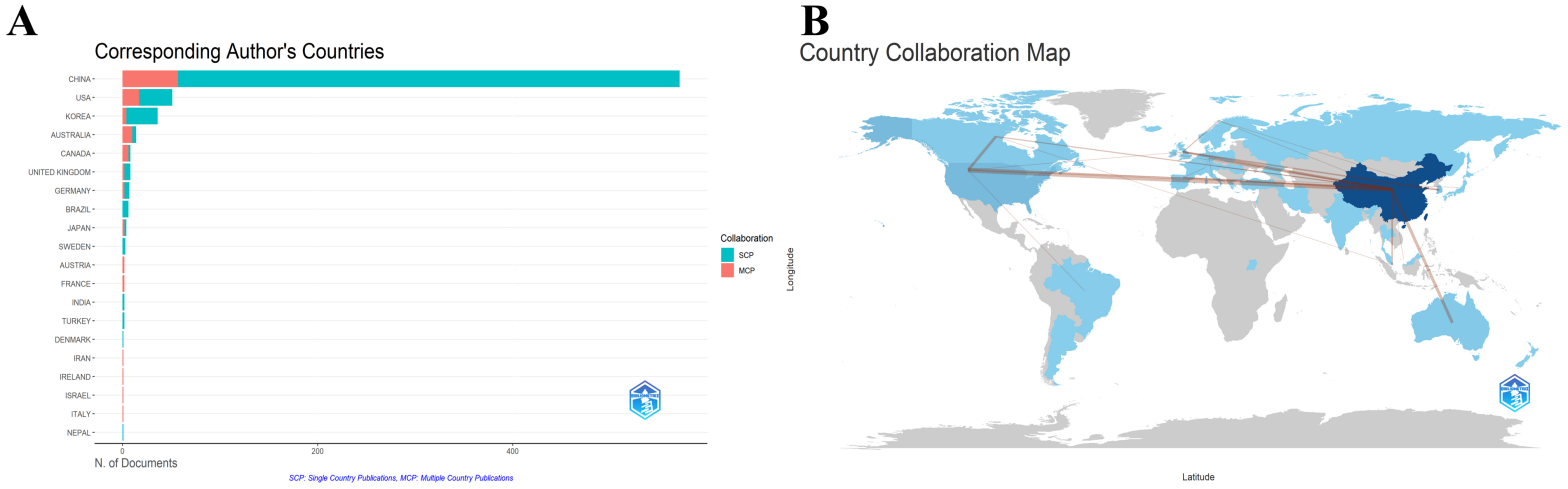
Countries/regions that have made contributions to the research field of traditional Chinese medicine in insomnia management. **(A)** Distribution of corresponding authors’ countries and cooperation. **(B)** Visualization map of cooperation among countries.

From 2005 to 2024, a total of 44 countries/regions have published articles related to the treatment of insomnia with traditional Chinese medicine. This study utilized Bibliometrix to analyze country/region and institutional collaboration networks. Table 3 presents the top 10 countries by the number of publications based on corresponding authors. China ranked first with 571 publications (77.9% of total output), far exceeding other countries. SCP (Single Country Publications) denotes publications authored exclusively by researchers from one country, MCP (Multinational Collaborative Publications) refers to publications co-authored by researchers from multiple countries, and MCP% represents the proportion of multinational collaborative publications (MCP% = Proportion of multinational collaborations). From an international collaboration perspective (Table 3; Figure 4), China produced the highest number of internationally collaborative publications (57). However, its MCP ratio was only 10%, significantly lower than that of the United States (33.3%) and Australia (71.4%). The scientific impact analysis (Table 4) revealed that publications from China received the highest total citations (8,337), followed by the United States (1,434) and Germany (1,329). However, China’s average citations per article (14.50) were markedly lower than those of Denmark (477.00), Germany (189.90), and Nepal (125.00). These findings indicate that while China dominates research output on the application of TCM therapies in insomnia, its lower average citation rate suggests room for improvement in international visibility and research quality. Furthermore, cross-regional collaborations in this field remain limited, highlighting the need for enhanced international cooperation in the future.

**Table 3:** The top 10 countries by the number of publications based on corresponding authors.

Country	Articles	Articles %	SCP	MCP	MCP %
China	571	77.9	514	57	10
USA	51	7	34	17	33.3
Korea	36	4.9	32	4	11.1
Australia	14	1.9	4	10	71.4
Canada	8	1.1	2	6	75
United kingdom	8	1.1	6	2	25
Germany	7	1	5	2	28.6
Brazil	6	0.8	6	0	0
Japan	4	0.5	2	2	50
Sweden	3	0.4	3	0	0

**Table 4:** Total Citations and Average Article Citations for National Publications.

Country	TC	Average article citations
China	8,337	14.50
USA	1,434	28.10
Germany	1,329	189.90
Korea	596	16.60
Denmark	477	477.00
Australia	414	29.60
United kingdom	166	20.80
Brazil	132	22.00
Nepal	125	125.00
Canada	106	13.20

**Figure 5:**
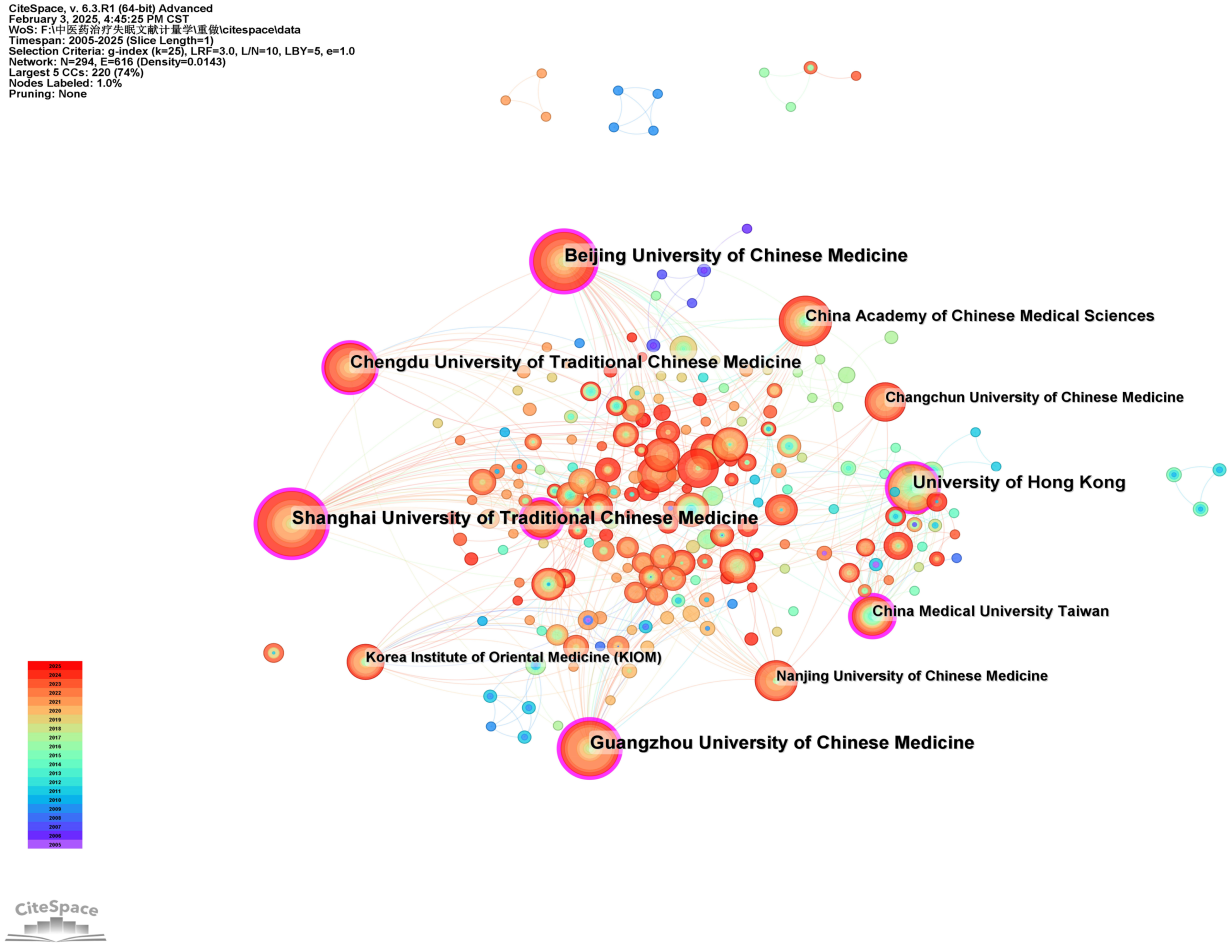
Network diagram of collaboration among institutions in TCM for insomnia management.

This study generated an institutional distribution map comprising 294 nodes and 616 connections using CiteSpace (Figure 5). As shown in Table 5, the top five institutions by publication volume are all based in China. Among these, Shanghai University of Traditional Chinese Medicine demonstrated the highest publication output in this field (58). Meanwhile, Table 6 reveals that Beijing University of Chinese Medicine ranked first in betweenness centrality (0.29) among the top 10 institutions. Notably, seven institutions exhibited centrality values exceeding 0.1, indicating their significant “bridging” roles in the collaboration network. These institutions served as critical intermediaries and coordinators in fostering inter-institutional cooperation. Of particular importance, Shanghai University of Traditional Chinese Medicine, Beijing University of Chinese Medicine, Guangzhou University of Chinese Medicine, and University of Hong Kong were consistently ranked among the top five institutions in both publication volume and centrality metrics. This dual prominence underscores their pivotal contributions to advancing research on the application of traditional Chinese medicine therapies in insomnia management.

**Table 5:** The top 10 Institutions by publication volume.

Rank	Institutions	Publications
1	Shanghai University of Traditional Chinese Medicine	58
2	Beijing University of Chinese Medicine	53
3	Guangzhou University of Chinese Medicine	50
4	University of Hong Kong	42
5	Chengdu University of Traditional Chinese Medicine	42
6	China Academy of Chinese Medical Sciences	31
7	China Medical University Taiwan	24
8	Nanjing University of Chinese Medicine	22
9	Changchun University of Chinese Medicine	21
10	Korea Institute of Oriental Medicine (KIOM)	20

**Table 6:** The betweenness centrality of the institutions.

Rank	Institutions	Centrality
1	Beijing University of Chinese Medicine	0.29
2	Guangzhou University of Chinese Medicine	0.22
3	Shanghai University of Traditional Chinese Medicine	0.20
4	China Medical University Taiwan	0.20
5	University of Hong Kong	0.14
6	Memorial Sloan Kettering Cancer Center	0.13
7	Chengdu University of Traditional Chinese Medicine	0.12
8	Harvard University	0.09
9	China Academy of Chinese Medical Sciences	0.08
10	Chinese University of Hong Kong	0.07

### 3.4 Analysis of journals

**Figure 6:**
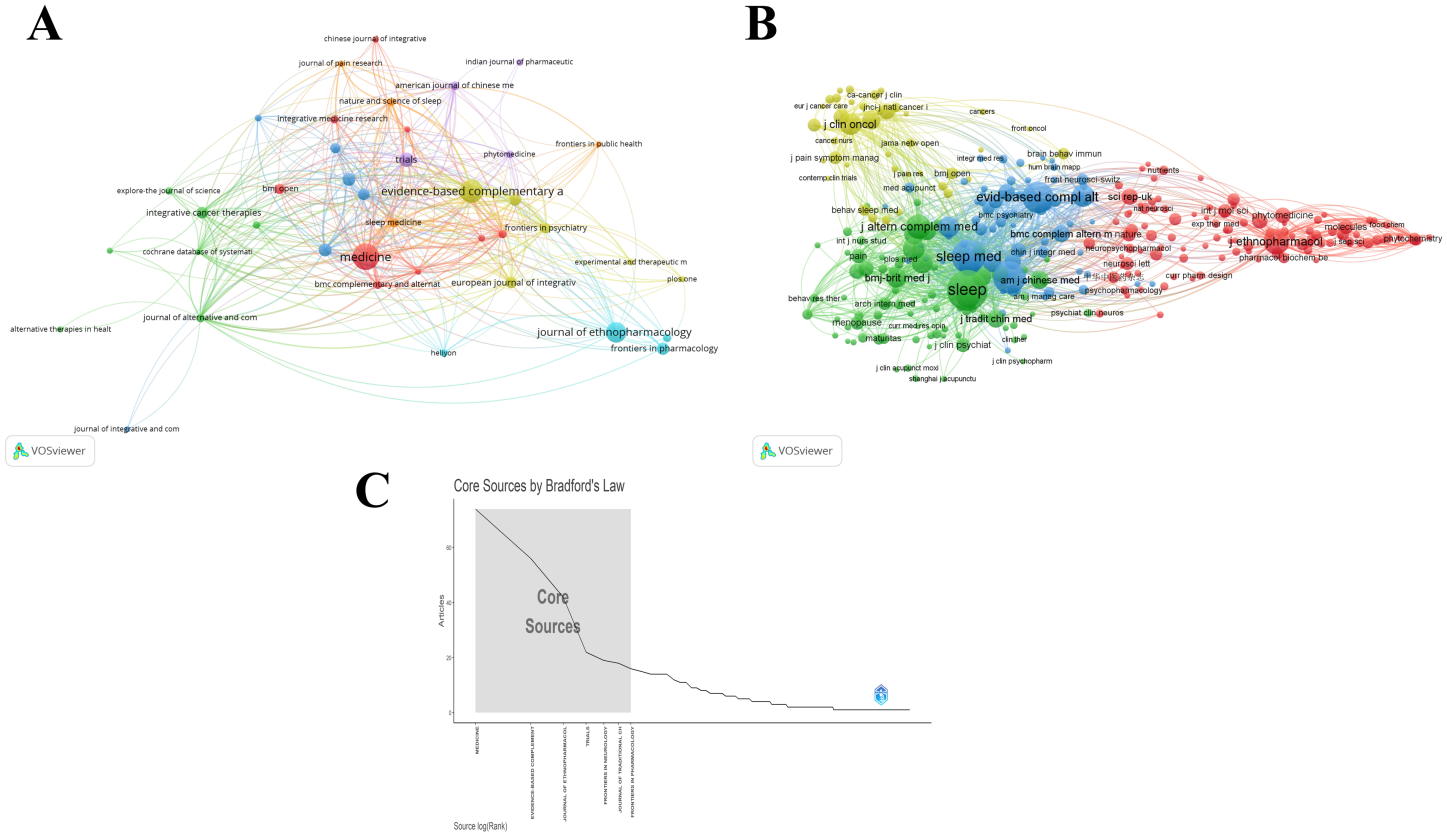
Analysis of journals in the field of TCM for insomnia management. **(A)** Visualization of cited journals. **(B)** Visualization of co-cited journals. **(C)** Seven core journals based on Bradford’s Law.

The journal distribution of publications was systematically processed and analyzed using VOSviewer. Our dataset encompassed 232 journals that have published articles on traditional Chinese medicine therapies for insomnia management. As can be seen from Figure 6, *Medicine*, *Evidence-Based Complementary and Alternative Medicine*, and *Journal of Ethnopharmacology* are journals with a relatively high number of published articles, while *Sleep*, *Sleep Medicine*, *Sleep Medicine Reviews*, and *Evidence-Based Complementary and Alternative Medicine* are journals with a relatively high number of co-citations. Table 7 presents the top 10 most productive journals, accompanied by their bibliometric indicators including total citations, impact factor, CiteScore, JCR ranking, and publisher information. The journal *Medicine* emerged as the most prolific publisher with 74 articles (10% of total publications), followed by *Evidence-Based Complementary and Alternative Medicine* (n = 56, 7.59%), *Journal of Ethnopharmacology* (*n* = 45, 6.1%), *Trials* (*n* = 22, 2.98%), and *Frontiers in Neurology* (*n* = 19, 2.57%). Notably, the impact factors of top 10 journals ranged from 1 to 5, with majority classified in JCR Q1/Q2 categories.

**Table 7:** The top 10 most productive journals, accompanied by their bibliometric indicators including total citations, impact factor, CiteScore, JCR ranking, and publisher information.

Rank	Journal	Publications	Frequency	IF (2023–2024)	CiteScore	JCR	Publishers
1	Medicine	74	316	1.4	2.80	Q2	Lippincott Williams and Wilkins Ltd.
2	Evidence-based Complementary and Alternative Medicine	56	1,007		3.50		Hindawi Publishing Corporation
3	Journal of Ethnopharmacology	45	1,071	4.8	10.30	Q1	Elsevier Ireland Ltd
4	Trials	22	182	2	3.80	Q3	BioMed Central
5	Frontiers in Neurology	19	54	2.7	4.90	Q2	Frontiers Media S. A.
6	Journal of Traditional Chinese Medicine	18	203	2	2.40	Q2	Journal of Traditional Chinese Medicine
7	Frontiers in Pharmacology	16	123	4.4	7.80	Q1	Frontiers Media S. A.
8	Integrative Cancer Therapies	15	188	2.9	4.80	Q2	SAGE Publications Inc.
9	Complementary Therapies in Medicine	14	295	3.3	8.60	Q1	Churchill Livingstone
10	Acupuncture in Medicine	14	218	2.4	4.70	Q2	BMJ Publishing Group

Table 8 presents the top 10 journals by citation and co-citation frequencies. The *Journal of Ethnopharmacology* recorded the highest citation count (1,071), while *Sleep* led in co-citations (1,008). Application of Bradford’s Law through bibliometrix analysis identified 7 core journals (Figure 6C). Despite its publication dominance, *Medicine* demonstrated relatively modest citation impact (316 total citations, ranking 5th), significantly lower than the top two journals exceeding 1,000 citations. Moreover, *Medicine* did not feature in the top 10 for co-citation counts. *Evidence-Based Complementary and Alternative Medicine* and *Journal of Ethnopharmacology* ranked consistently in top 10 across publication volume, citations, and co-citations. However, it should be noted that *Evidence-Based Complementary and Alternative Medicine* was excluded from SCIE directory in 2023. The Q1-ranked *Journal of Ethnopharmacology* (IF = 4.8) demonstrated robust academic influence with 45 publications accumulating 1,071 citations and 413 co-citations in our dataset. Consequently, *Journal of Ethnopharmacology* and *Medicine* are likely the most influential journals in the application of TCM therapies for insomnia.

**Table 8:** The top 10 journals by citation and co-citation frequencies.

Rank	Cited journal	Frequency	Rank	Co-cited journal	Frequency
1	Journal of Ethnopharmacology	1,071	1	Sleep	1,008
2	Evidence-based Complementary and Alternative Medicine	1,007	2	Sleep Medicine	680
3	Sleep Medicine Reviews	476	3	Sleep Medicine Reviews	653
4	Sleep Medicine	398	4	Evidence-based Complementary and Alternative Medicine	591
5	Medicine	316	5	Journal of Ethnopharmacology	413
6	Journal of Alternative and Complementary Medicine	300	6	Journal of Alternative and Complementary Medicine	368
7	Complementary Therapies in Medicine	295	7	Zhongguo zhen jiu	335
8	American Journal of Chinese Medicine	258	8	PLOS ONE	306
9	BMC Complementary Medicine and Therapies	257	9	Journal of Clinical Oncology	289
10	Acupuncture in Medicine	218	10	Cochrane Database of Systematic Reviews	285

### 3.5 Analysis of cited references

**Figure 7:**
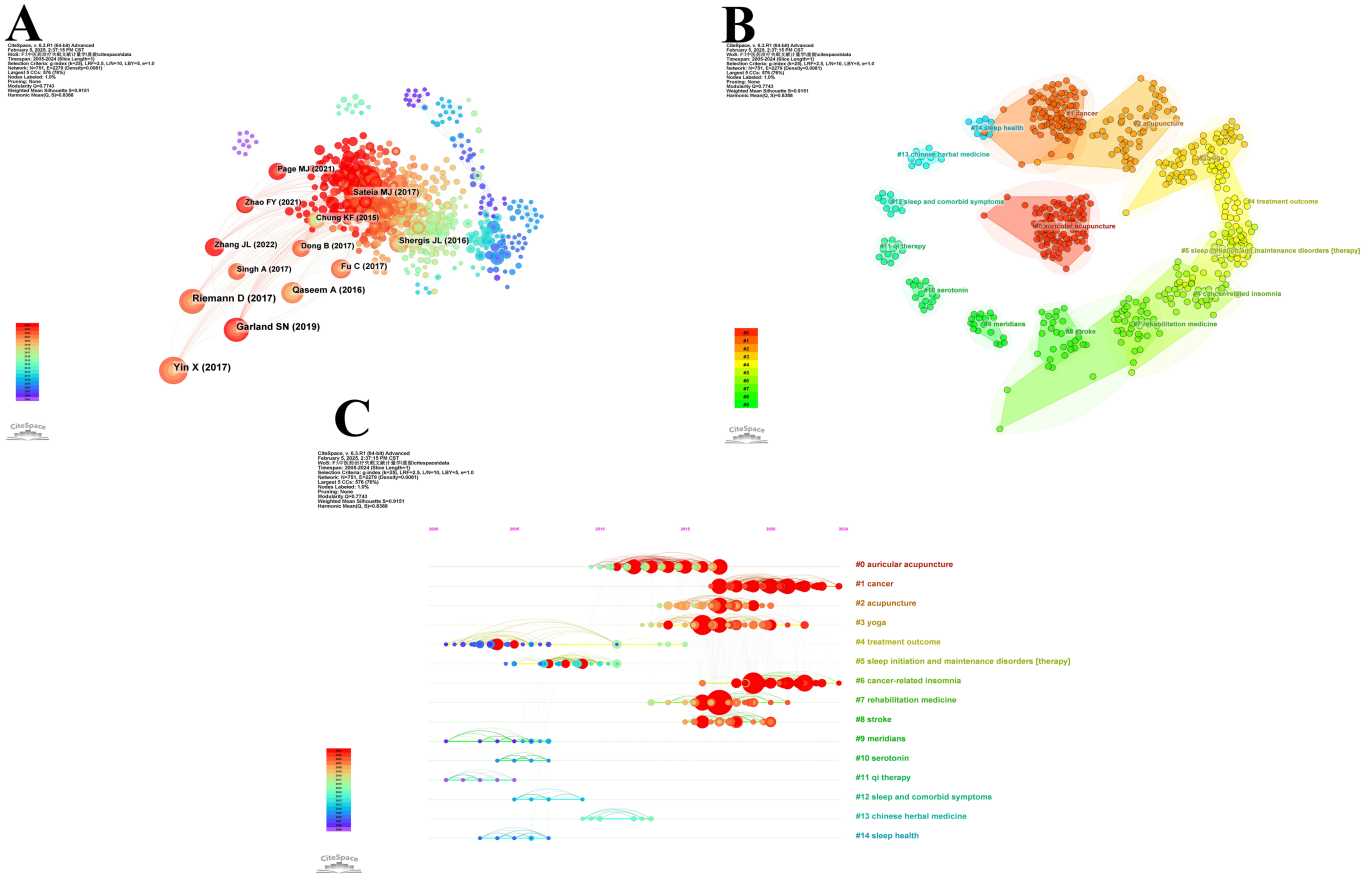
Analysis of cited references in TCM for insomnia management. **(A)** Network diagram of cited references. **(B)** Cluster analysis diagram of cited references. **(C)** Cluster timeline of cited references.

The citation network was constructed using CiteSpace, generating a visualization map comprising 751 nodes and 2,279 links (Figure 7A). Table 9 lists the top 13 cited publications in this field. The study by Yin, Gou M, Xu J, et al. (2017), titled “Efficacy and safety of acupuncture treatment on primary insomnia: a randomized controlled trial,” ranked first with 47 citations. Red nodes in the network denote recently cited references, with three of the top-cited articles published post-2020. Notably, the majority of these articles (10) were published in JCR Q1 journals. Content analysis revealed that the highly cited reference predominantly focused on acupuncture interventions for insomnia. Specifically, four studies were randomized controlled trials evaluating acupuncture efficacy, four were systematic reviews synthesizing evidence on acupuncture, and four represented clinical guidelines related to insomnia management. Only one systematic review investigated herbal medicine for insomnia. This distribution underscores the current research emphasis on acupuncture as the primary TCM modality explored in insomnia studies, with limited attention to other traditional therapeutic approaches.

**Table 9:** The top 13 cited publications

Rank	References	Representative author (publication year)	Frequency	journal	IF(2023-2024)	JCR	DOI
1	Efficacy and safety of acupuncture treatment on primary insomnia: a randomized controlled trial	Yin X, Gou M, Xu J, et al(2017)	47	Sleep Medicine	3.8	Q1	10.1016/j.sleep.2017.02.012
2	European guideline for the diagnosis and treatment of insomnia	Riemann D, Baglioni C, Bassetti C, et al(2017)	40	Journal of Sleep Research	3.4	Q2	10.1111/jsr.12594
3	Acupuncture Versus Cognitive Behavioral Therapy for Insomnia in Cancer Survivors: A Randomized Clinical Trial	Garland SN, Xie SX, DuHamel K, et al(2019)	38	JNCI-Journal of the National Cancer Institute	10	Q1	10.1093/jnci/djz050
4	Management of Chronic Insomnia Disorder in Adults: A Clinical Practice Guideline From the American College of Physicians	Qaseem A, Kansagara D, Forciea MA, Cooke M, Denberg TD(2016)	29	Annals of Internal Medicine	19.6	Q1	10.7326/M15-2175
5	Acupuncture Improves Peri-menopausal Insomnia: A Randomized Controlled Trial	Fu C, Zhao N, Liu Z, et al(2017)	23	Sleep	5.3	Q1	10.1093/sleep/zsx153
6	A systematic review of acupuncture for sleep quality in people with insomnia	Shergis JL, Ni X, Jackson ML, et al(2016)	22	Complementary Therapies in Medicine	3.3	Q1	10.1016/j.ctim.2016.02.007
7	Clinical Practice Guideline for the Pharmacologic Treatment of Chronic Insomnia in Adults: An American Academy of Sleep Medicine Clinical Practice Guideline	Sateia MJ, Buysse DJ, Krystal AD, Neubauer DN, Heald JL(2017)	21	Journal of Clinical Sleep Medicine	3.5	Q1	10.5664/jcsm.6470
8	Acupuncture for cancer-related insomnia: A systematic review and meta-analysis	Zhang J, Zhang Z, Huang S, et al(2022)	20	Phytomedicine	6.7	Q1	10.1016/j.phymed.2022.154160
9	Acupuncture for residual insomnia associated with major depressive disorder: a placebo- and sham-controlled, subject- and assessor-blind, randomized trial	Chung KF, Yeung WF, Yu YM, et al(2015)	18	Journal of Clinical Psychiatry	4.5	Q1	10.4088/JCP.14m09124
10	The Efficacy of Acupuncture for Treating Depression-Related Insomnia Compared with a Control Group: A Systematic Review and Meta-Analysis	Dong B, Chen Z, Yin X, et al(2017)	18	Biomed Research International	2.6	Q3	10.1155/2017/9614810
11	Can acupuncture improve objective sleep indices in patients with primary insomnia? A systematic review and meta-analysis	Zhao FY, Fu QQ, Kennedy GA, et al(2021)	18	Sleep Medicine	3.8	Q1	10.1016/j.sleep.2021.01.053
12	The PRISMA 2020 statement: an updated guideline for reporting systematic reviews	Page MJ, McKenzie JE, Bossuyt PM, et al(2021)	18	BMJ-British Medical Journal	93.7	Q1	10.1136/bmj.n71
13	Treatment of Insomnia With Traditional Chinese Herbal Medicine	Singh A, Zhao K(2017)	18	International Review of Neurobiology			10.1016/bs.irn.2017.02.006

In this study, we employed CiteSpace to conduct a cluster analysis of the cited references, resulting in the identification of 14 distinct clusters (Figure 7B). The largest cluster, #0 auricular acupuncture (size = 101, Silhouette = 0.917), was the most prominent among the 14 clusters. Following clustering analysis of the cited references, the quantitative evaluation revealed a Modularity Q-value of 0.7743 and a weighted mean silhouette coefficient (S) of 0.9151. These metrics significantly surpass the established thresholds for cluster validity (Q > 0.3 and S > 0.7), demonstrating a clustering structure with high reliability in the classification outcomes. As illustrated in Figure 7C, #4 treatment outcome emerged as the earliest hotspot cluster, while #1 cancer and #6 cancer-related insomnia represent the most recent hotspot clusters, indicating a growing emphasis on insomnia related to cancer in the field.

**Figure 8:**
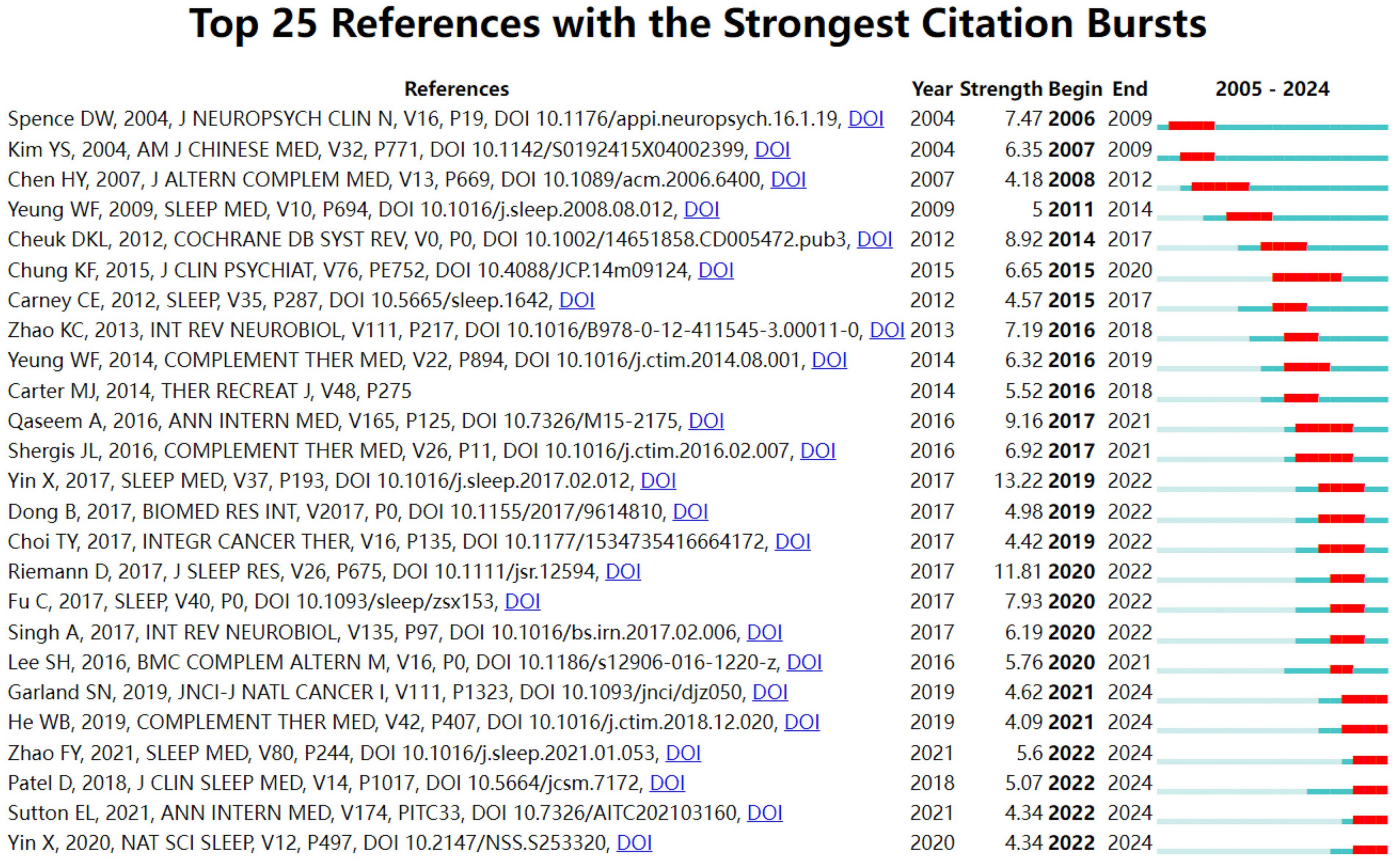
Top 25 references with the strongest citation bursts in TCM for insomnia management.

This study utilized CiteSpace temporal clustering analysis (Number of States: 2, Minimum Duration: 2) to identify the top 25 references with the strongest citation bursts from 2005 to 2024 (Figure 8). The article by Yin et al. (2017) titled “Efficacy and safety of acupuncture treatment on primary insomnia: a randomized controlled trial” (*Sleep Medicine*, strength: 13.22) ranked first, with its citation burst lasting from 2019 to 2022. Additionally, six references on acupuncture and electroacupuncture showed continuous citation bursts until 2024. These findings demonstrate that acupuncture and electroacupuncture interventions for insomnia management (particularly addressing cancer-related and depression-related insomnia) have emerged as a focal research area in traditional Chinese medicine applications for insomnia.

### 3.6 Analysis of keywords

**Figure 9:**
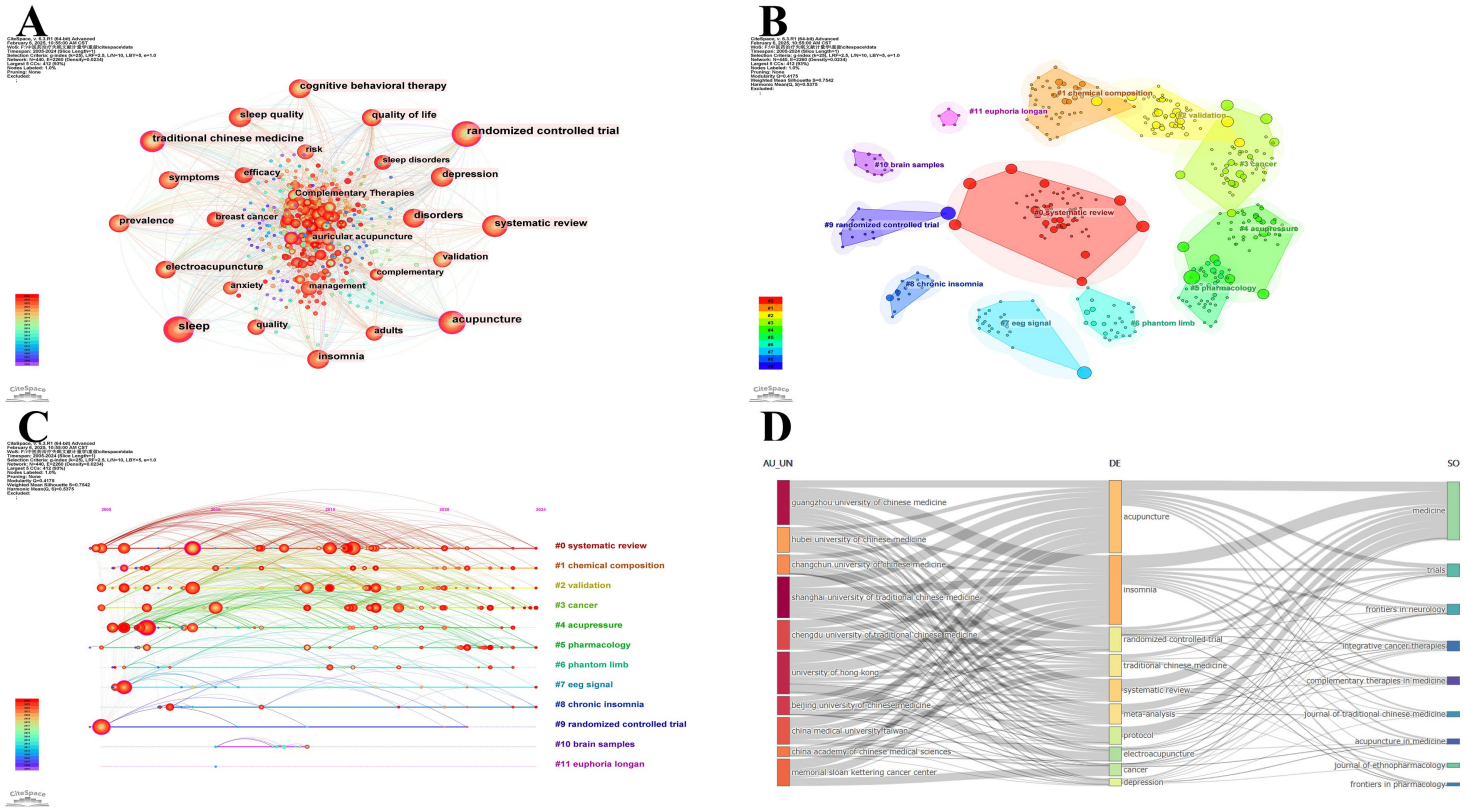
Analysis of keywords in TCM for insomnia management. **(A)** Network diagram of keywords. **(B)** Cluster analysis diagram of keywords. **(C)** Cluster timeline of keywords. **(D)** A three-field plot delineating the interconnections among keywords, institutions, and journals.

Keyword co-occurrence mapping reveals research hotspots in the field while burst keywords indicate emerging frontiers. To ensure analytical rigor, we first manually consolidated synonymous terms such as “acupuncture” with “acupuncture therapy,” “chemical composition” with “chemical constituents,” and “cognitive behavioral therapy” with “cognitive behavior therapy.” Using CiteSpace, we constructed a keyword co-occurrence network comprising 440 nodes and 2,260 links (Figure 9A). As shown in Table 10, we listed the top 10 keywords by occurrence frequency with “sleep” being the only term exceeding 100 occurrences. Table 11 shows that the keywords with significant betweenness centrality (≥0.1) are “randomized controlled trial” (0.13) “acupuncture” (0.13) “traditional Chinese medicine” (0.13) “brain” (0.12)

and “sleep” (0.10). The integration of frequency metrics and centrality analysis identifies “sleep,” “randomized controlled trial,” “traditional Chinese medicine,” and “acupuncture” as pivotal research foci in traditional Chinese medicine applications for insomnia treatment.

**Table 10:** The top 10 keywords by occurrence frequency.

Rank	Keyword	Frequency
1	Sleep	106
2	Randomized controlled trial	92
3	Acupuncture	86
4	Traditional Chinese medicine	68
5	Systematic review	66
6	Disorders	62
7	Cognitive behavioral therapy	60
8	Prevalence	58
9	Insomnia	55
10	Depression	54

**Table 11:** Keywords with intermediate centrality greater than or equal to 0.1.

Rank	Keyword	Centrality
1	Randomized controlled trial	0.13
2	Acupuncture	0.13
3	Traditional Chinese medicine	0.13
4	Brain	0.12
5	Sleep	0.10

Using CiteSpace, we performed co-occurrence keyword clustering analysis and identified 11 distinct thematic clusters (Figure 9B). Our analysis yielded robust results (Q = 0.4175, S = 0.7542), confirming the structural validity and reliability of the clustering network. Thematic categorization of clusters revealed four research dimensions: Research Methodologies: #0 systematic review, #2 validation, #9 randomized controlled trial; Basic Research: #1 chemical composition, #5 pharmacology, #7 EEG signal, #10 brain samples; Therapeutic Interventions: #4 acupressure, #11 *Euphoria longan*; Symptomatology and Comorbidity: #3 cancer, #6 phantom limb, #8 chronic insomnia. The timeline overlay analysis (Figure 9C) demonstrated persistent scholarly attention to clusters #0 systematic review, #1 chemical composition, #2 validation, #3 cancer, #5 pharmacology, #7 EEG signal, and #8 chronic insomnia, suggesting their ongoing evolution and potential to generate novel research trajectories.

Figure 9D presents a three-field plot delineating the interconnections among keywords, institutions, and journals in this domain. Institution-specific keyword preferences were observed: The University of Hong Kong exhibited pronounced interest in “electroacupuncture,” whereas Memorial Sloan Kettering Cancer Center focused predominantly on “cancer.” Notably, Guangzhou University of Chinese Medicine demonstrated the broadest research coverage across all 10 keywords compared to other institutions. At the journal level, *Medicine* emerged as the most prolific contributor to these keywords. While *Trials* published more articles associated with “acupuncture” than *Frontiers in Neurology*, it showed limited engagement with “systematic review,” “meta-analysis,” and “cancer.”

**Figure 10:**
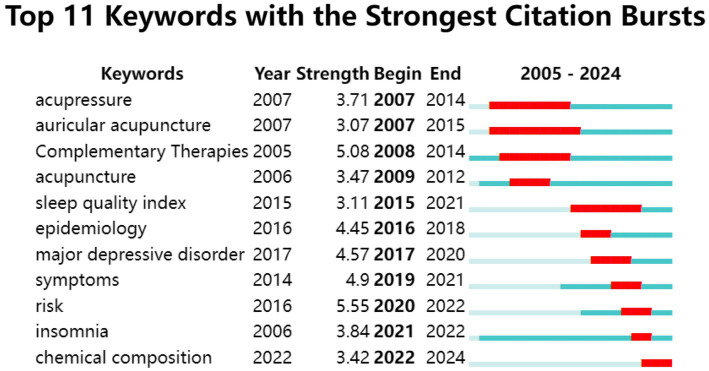
Top 11 keywords with the strongest citation bursts in TCM for insomnia management.

Using CiteSpace (parameters: Number of States = 2, Minimum Duration = 2), we identified the top 11 keywords with the strongest citation bursts from 2005 to 2024 (Figure 10). The keyword “risk” (burst strength: 5.55) displayed the highest intensity, surging from 2020 to 2022. “Auricular acupuncture” and “acupressure” were the earliest burst keywords (emerging in 2007), with the former maintaining the longest duration (9 years). Notably, “chemical composition” (2022–2024) represents the most recent persistent burst keyword, suggesting its status as an emerging research frontier and potential future direction.

## 4 Discussion

### 4.1 Summary of the main findings

This study used bibliometric tools to analyze the research dynamics of traditional Chinese medicine in treating insomnia from 2005 to 2024 and identified three critical developments. Firstly, annual publications exhibited sustained growth from 2016 onward, culminating in 99 articles by 2021 with persistently high productivity through 2024. Secondly, cumulative scholarly outputs totaled 738 publications by the study’s endpoint, accumulating 13,629 citations. Thirdly, quantitative metrics confirmed robust academic impact, evidenced by an average citation rate of 18.47 per article. The substantial growth in both publication outputs and citation metrics clearly demonstrates the escalating academic attention this field has attracted in recent years.

China’s research leadership is evidenced by 571 publications (77.4% of total outputs), confirming its pivotal role in advancing TCM-based insomnia studies. Among the top 10 productive institutions, nine were Chinese. Despite these institutions formed a robust domestic collaboration network, their international engagement remained limited as reflected by a multinational collaboration percentage of only 10%, which was significantly lower than leading countries such as the United States (33.3%) and Australia (71.4%). This intensive localization-sparse internationalization dual structure likely contributed to suboptimal citation impact (14.50 citations/article), reflecting a quantity-quality imbalance in Chinese research outputs. These findings highlight the necessity for China to prioritize enhancing research quality through strengthened global collaboration while sustaining publication momentum.

In terms of publication productivity, the top 10 authors accounted for 22.9% of total outputs. Yeung, Wing-Fai from Hong Kong Polytechnic University dominates as the most prolific contributor and third most co-cited scholar. His collaboration network with Chung, Ka Fai and Zhang, Zhang-Jin formed the productivity core, contributing to 12.26% of China’s total outputs. Yeung’s research spans three core areas: sleep medicine, traditional Chinese medicine, and mental health/psychological interventions, encompassing pain management and COVID-19-related studies. In applying traditional TCM therapies to insomnia, his work focuses on acupuncture (34), electroacupuncture (35, 36), auricular acupuncture (37, 38), and acupressure (39). As early as 2009, he reported that electroacupuncture demonstrated superior short-term efficacy compared to sham acupuncture for primary insomnia (35). Furthermore, in 2023, his investigation advanced clinical applications by demonstrating combined electroacupuncture/auricular acupressure benefits for breast cancer patients with chemotherapy-induced insomnia (40).

In terms of journal contributions, *Medicine* (74 publications, 316 citations) and *Journal of Ethnopharmacology* (45 publications, 1,071 citations) emerged as the most influential journals. However, *Medicine* demonstrated significantly lower academic impact, likely related to its multidisciplinary scope compared to *Journal of Ethnopharmacology*, which specializes in traditional pharmacological mechanism research. Bradford’s Law identified seven core journals with JCR categories concentrated in Q1-Q2, indicating increasing acceptance of TCM-related insomnia research by mainstream medical journals.

### 4.2 Hotspots and trends

#### 4.2.1 Strategies and mechanisms of acupuncture in treating insomnia

Through analysis of citation counts, clustering, and burst detection, as well as keyword frequency, centrality, and burst patterns, we identified acupuncture as a prominent research hotspot. Insomnia ranks among the most responsive conditions to acupuncture therapy (41). Multiple modalities including manual acupuncture (21), electroacupuncture (20), auricular acupuncture (42) have demonstrated efficacy, with scalp acupuncture potentially being the most effective single approach (43), while acupoints catgut embedding shows particular advantages in combined regimens (22).

Recent studies highlight advancements in both clinical validation and mechanistic exploration. Acupuncture outperforms sham/placebo acupuncture in improving PSQI, Insomnia Severity Index, total sleep time, sleep onset latency, wake after sleep onset, and sleep efficiency, with sustained effects (44). Compared to Western pharmaceuticals, acupuncture demonstrates fewer adverse effects while achieving superior PSQI improvements (45), with enhanced efficacy observed after 3 weeks (46). It also reduces dependency risks associated with benzodiazepines and non-benzodiazepine hypnotics (47, 48). Mechanistic studies reveal central nervous system modulation as a primary pathway, fMRI evidence shows acupuncture modulates brain functional connectivity in the default mode network (frontal lobe/precuneus) (49), locus coeruleus (50), hypothalamus (51), and emotional network (left amygdala-thalamus) (52). EEG studies demonstrate that acupuncture at GV20 (Baihui) increases *α*-wave power and decreases *β*-wave power in sleep-deprived rats, exerting sedative effects (53). Clinical investigations further confirm acupuncture downregulates steady-state visual evoked potentials in frontal and occipital cortices of insomnia patients with emotional disorders, effectively inhibiting abnormal brain electrical excitability (54). Emerging evidence links gut microbiota dysbiosis to insomnia pathogenesis, acupuncture interventions targeting the microbiota-gut-brain axis (MGBA) show promise in improving sleep quality (55). Hong et al. (56) demonstrated acupuncture remodels gut microbiota (increasing Lactobacillus, decreasing Clostridium XIVb/Lachnospiracea incertae sedis, and balancing Firmicutes/Bacteroidetes ratio) to regulate immune responses in PCPA-induced insomnia mice. An MGBA-targeted acupuncture protocol (DU20, DU24, RN12, PC6, HT7, ST36, SP6) has been proposed to improve sleep via microbiota-neuro-immune network modulation (57).

Notably, current acupuncture research faces limitations in randomized controlled trial design, blinding implementation, and large-scale multicenter validation, resulting in suboptimal evidence quality. Enhancing methodological rigor and establishing standardized research paradigms are critical for improving international recognition within evidence-based medicine frameworks. Collectively, acupuncture’s validated efficacy, multidimensional mechanisms, and favorable safety profile position it as a prioritized therapeutic strategy in TCM insomnia research.

#### 4.2.2 Pharmacological constituents and mechanisms of TCM in insomnia treatment

Through clustering and burst analysis of keywords, “chemical composition” was identified as a persistent research hotspot. This indicates that the analysis of chemical components in Chinese medical herbs has become a central research focus for anti-insomnia studies. Investigations have identified *Suanzaoren* (*Ziziphi Spinosae*), *Fuling* (*Poria*), *Yejiaoteng* (*Caulis P. multiflori*), *Gancao* (*Radix Glycyrrhizae*), and *Baishao* (*Radix P. alba*) as commonly used herbal ingredients in treating diverse insomnia conditions (58). Among these, Z*iziphi Spinosae Semen* has emerged as a research focus due to its significant sedative effects, establishing a comprehensive “component identification-pharmacodynamic verification-mechanism analysis” framework.

Herbal medicines possess a complex and diverse chemical profile. With the advancement of analytical technologies, particularly liquid chromatography, the systematic identification of bioactive compounds with therapeutic efficacy against insomnia has become increasingly feasible. For example, Bosai He et al. (59) quantified key insomnia-treating components (spinosin, mangiferin, ferulic acid) in Suanzaoren Decoction using ultra-fast liquid chromatography/tandem mass spectrometry (UFLC-MS/MS). Hongjuan Li et al. (60) achieved >98% purity and 72.6% recovery of spinosin through HPD-300 macroporous resin combined with high-performance liquid chromatography, thereby providing a solid foundation for the standardized and large-scale production of bioactive compounds from TCM for insomnia treatment. To date, over 150 compounds have been identified in Ziziphi Spinosae Semen, including terpenoids, alkaloids, flavonoids, fatty acids, volatile oils, and polysaccharides. Hypnotic effects primarily derive from triterpenoid saponins (e.g., jujuboside A/B/C), flavonoids, and alkaloids (61). In terms of pharmacodynamic validation, single-component extracts (saponins/fatty acids) and herb pair extracts (spinosin and senegenin) significantly prolonged sleep duration and shortened sleep latency in mice (62, 63).

In mechanistic studies of TCM chemical components, animal experiments have demonstrated that Ziziphi Spinosae Semen extract modulates GABAergic systems by upregulating GABAARα1/GABAARγ2 expression and GABA/5-HT levels while downregulating Glu/DA in hypothalamus/hippocampus (64). It also restores gut-brain axis function by balancing phenylalanine/histidine metabolism and gut microbiota (increasing short-chain fatty acids, inhibiting pro-inflammatory bacteria), ultimately enhancing sleep quality (63).

Our study also reveals that “pharmacology” represents a continuously evolving keyword cluster, with network pharmacology emerging as a pivotal tool for deciphering ingredient-target-pathway relationships in Chinese medical herbs and offering novel insights into the mechanisms of action underlying their complex components. Network pharmacology studies have identified 11 active compounds in Ziziphus spinosa, including swertisin, jujuboside A, and spinosin. These compounds target 48 core proteins, such as TNF, IL-6, and AKT1. They regulate dopaminergic synapses and the cAMP signaling pathway, clarifying the mechanisms by which Ziziphus spinosa improves insomnia (65). Suanzaoren Decoction with 138 components (kaempferol, quercetin) act on IL-6/AKT1/TNF/VEGFA to modulate neuroactive ligand-receptor interactions (DA/5-HT/orexins/GABA), serotonergic synapse, and PI3K/AKT pathways (66). Network pharmacology has revealed tha both mono- and multi-herb formulations appear to exert anti-insomnia effects via inflammation-neurotransmitter regulation.

In summary, advancements in analytical and omics technologies will likely uncover additional bioactive compounds and synergy networks, driving systematic and international development of anti-insomnia Chinese medical herbs research.

#### 4.2.3 TCM in the treatment of insomnia caused by depression and cancer

Bibliometric analysis incorporating citation counts, clustering, burst detection, and keyword co-occurrence identified “depression-related insomnia” and “cancer-related insomnia” as emerging research frontiers, highlighting growing interest in comorbid and secondary insomnia. The International Classification of Sleep Disorders, Third Edition (ICSD-3) subsumes secondary insomnia and comorbid insomnia under the umbrella of chronic insomnia disorder (67). In clinical practice, therapeutic strategies for disease-associated insomnia predominantly emphasize primary disease management (68). However, accumulating evidence reveals bidirectional relationships between insomnia and comorbid conditions (69), including malignancies (70) and depressive disorders (71). Given that sleep disturbances may exacerbate disease progression, targeted interventions not only ameliorate insomnia symptoms but also synergistically enhance primary disease outcomes (67). As the first-line treatment for insomnia, CBT-I demonstrates equivalent effectiveness in managing comorbid insomnia, exhibits particularly positive therapeutic effects on psychiatric comorbidities (10), and is recommended as the preferred intervention for patients with comorbid insomnia and depression (72). Among Traditional Chinese Medicine interventions, acupuncture emerges as the most frequently reported therapy for managing disease-associated insomnia, with clinical evidence supporting its application in depression-related (73), cancer-related (74), perimenopausal (75), post-stroke (76), and chronic pain-associated insomnia (77).

Depression imposes a significant global health burden, ranking as the second leading cause of years lived with disability (YLDs) in 2021 (78). Depression and insomnia are closely related, and among patients with chronic insomnia, depression seems to show the highest comorbidity tendency (79). In major depressive episodes, 85.2% of patients experience insomnia (80). The negative impact of the comorbidity of the two on quality of life is significantly greater than the sum of the effects of the two conditions existing separately (71). Potential mechanisms underlying the interaction between insomnia and depression may include inflammatory activation, imbalance of the monoaminergic system, circadian rhythm disorders, and certain genetic factors (81). Research on the treatment of depression-related insomnia with traditional Chinese medicine mainly focuses on acupuncture. A high-quality trial showed electroacupuncture significantly and sustainably improved sleep quality versus conventional care or sham acupuncture (82). Low-to-moderate evidence supports acupuncture as an alternative or adjunct to pharmacotherapy for symptom relief (83). The improvement of insomnia and depressive symptoms by acupuncture may be achieved by increasing serum corticosterone and decreasing serum 5-HT (84). However, some studies have shown that acupuncture has no significant effect on the total PSQI score of patients with severe depression (85). There are relatively few studies on the treatment of depression-related insomnia with Chinese medical herbs and decoctions. However, Chinese medical herbs and decoctions commonly used for insomnia have been reported to be effective in treating depression. For example, Magnoflorine (MAG) and Spinosin (SPI) in Ziziphi Spinosae Semen have antidepressant effects (86), and the Suanzaoren Decoction may exert its antidepressant effect by jointly regulating the TLR4/MyD88/NF-κB pathway and the Wnt/*β*-catenin pathway (87).

Cancer-related insomnia is an increasingly concerned insomnia subtype. According to demographic data, nearly 20 million new cancer cases were reported globally in 2022, with projections indicating a rise to 35 million by 2050 (88). The prevalence of insomnia among cancer patients ranges from 42 to 62%, with 12–33% meeting clinical diagnostic criteria for insomnia (89–91). High-incidence cancer-related psychological symptoms (CRPS), such as insomnia, are associated with increased cancer morbidity and mortality (92). The ESMO Clinical Practice Guidelines recommend CBT-I as the standard care for cancer survivors (93). Acupuncture and CBT-I both relieve insomnia in cancer patients. While CBT-I shows stronger effects, acupuncture adds rapid cancer pain relief alongside sleep improvement (94, 95). The most effective acupuncture therapies may combine auricular therapy with moxibustion or traditional acupuncture, with auricular therapy primarily enhancing sleep through vagus nerve stimulation (96). Breast cancer is the subtype with the most research on acupuncture for cancer-related insomnia (97, 98). Breast cancer has the second-highest incidence rate after lung cancer, and breast cancer patients are more prone to insomnia than other cancer patients and the general population. More than a quarter of them develop new insomnia during chemotherapy, and the symptoms are more likely to persist (88). A high-quality RCT has shown that acupuncture can improve chemotherapy-related insomnia in breast cancer patients in both short-term and long-term follow-ups, reducing or even replacing the use of sleeping pills (40). In addition, it has been reported that Tai Chi and Qigong can improve subjective sleep problems in breast cancer patients (88). Early and short-term use of traditional Chinese herbal products such as Tian-wang-bu-xin-dan and Suanzaoren Decoction to relieve insomnia may increase the 5-year survival rate of breast cancer patients (99).

This study confirms “acupuncture” as a core hotspot, aligning with Yi Huang’s team (28) and Wenya Pei’s team (27), indicating that acupuncture is the most widely studied therapy in the field of TCM for treating insomnia. Notably, Junxin Wang et al. (29), focusing on TCM nursing techniques, conclude that auricular therapy is a promising nursing intervention for insomnia. This divergence in perspectives arises from the difference in research scope (therapy vs. nursing), reflecting the specialization within the TCM discipline. Yi Huang et al. (28) highlight “secondary insomnia” and “co-morbid insomnia,” and this study extends this focus to cancer- and depression-related subtypes. Specifically, this study found that breast cancer-related insomnia is the most researched subtype of cancer-related insomnia treated by TCM. Although Junxin Wang et al. (29) mentioned comorbidities such as breast cancer, anxiety, and depression, they emphasized that “hemodialysis complicated with insomnia” is one of the main research trends in TCM nursing techniques. Regarding herbal medicine, Qianyan Wu et al. (30) identify “oral herbs combined with acupuncture” as a recent hotspot, similar to the conclusions of this study. However, Qianyan Wu et al. (30) focused on discussing the safety and dosage form of Chinese herbal medicine, this study centered on the hotspot of “chemical composition” to deeply analyze the active ingredients, pharmacological mechanisms, and network pharmacology pathways of Chinese herbal medicine. This may be attributed to their use of dosage form-related search terms such as “Powder” and “Capsule” in their search strategy, and their inclusion criteria were clinical studies.

## 5 Limitations

Firstly, the data were exclusively sourced from English-language literature in the Web of Science database. The WoSCC offers a comprehensive set of bibliometric indicators (e.g., h-index, citation counts) and structured metadata, which are crucial for conducting thorough bibliometric analyses. Its standardized format enables effective cross-study comparisons. By restricting our data source to WoS, we ensure the replicability of our study using a widely recognized and accessible database. Expanding the data sources to include multiple databases could introduce variability due to overlapping indexing and inconsistencies. Including additional databases such as Scopus and PubMed would complicate data extraction and deduplication, potentially increasing the risk of errors. Additionally, some databases, like PubMed, do not allow the export of full records and cited references. Given the trade-off between data breadth and depth, we prioritize data depth. Furthermore, in medical research, there is considerable overlap between records indexed in WoS and those found in other databases like PubMed. Therefore, we are confident that the results of our study are methodologically robust and reliable. Secondly, due to temporal constraints, the long-term impact of 2024 publications has not yet fully emerged, and exclusion of 2025 articles may weaken forward-looking discipline assessments. Thirdly, despite manually merging synonymous terms, complete elimination of synonym recognition bias remains challenging. Fourthly, this study did not limit the diagnostic criteria for the included clinical research literature, which may lead to research heterogeneity. However, as a bibliometric study focusing on macro-trends, its core lies in analyzing the overall characteristics of the field rather than the internal validity of individual studies. Therefore, the above limitations have a limited impact on the overall conclusions. Additionally, although the search strategy of this study was systematically designed and adhered to bibliometric research norms, it was not evaluated by a professional librarian, which may introduce potential biases in terms of keyword selection or database coverage. The research team has minimized such impacts through dual independent retrieval and cross-checking. Nonwithstanding these limitations, this investigation provides timely bibliometric evidence for the field of traditional Chinese medicine in insomnia treatment through systematic review of research trajectories and scientific impact distribution over the past two decades. The methodological framework established herein offers valuable reference for subsequent studies integrating multi-source data.

## 6 Conclusion

This study systematically reveals the research trends of traditional Chinese medicine in treating insomnia from 2005 to 2024 through bibliometrics. As a common health issue in modern society, insomnia is not only an independent sleep disorder but also a potential risk factor for various diseases. Compared to internationally recognized CBT-I, TCM demonstrates unique advantages in reducing medication dependence and managing complex comorbid conditions. Since 2016, TCM therapies have received increasing attention in the field of insomnia, especially acupuncture, chemical components, and the management of cancer-related insomnia and depression-related insomnia. Research orientation is transitioning from clinical efficacy assessment to in-depth mechanistic exploration, with network pharmacology emerging as a burgeoning trend for deciphering the action targets of active components in traditional Chinese medicine. While China remains the predominant contributor through institutional collaborative networks, international cooperation and research quality require further enhancement. Future directions should prioritize: (1) Mechanistic investigations integrating modern technologies to decipher acupuncture and herbal regulatory pathways. (2) Expanded exploration of non-acupuncture therapies. (3) Strengthened interdisciplinary and global collaborations to conduct multi-center clinical trials with enhanced methodological quality. (4) Development of stratified intervention protocols for comorbid insomnia in cancer and depression, alongside preventive strategies aligned with the “preventive treatment” principle. (5) Evidence translation through publication of rigorous RCTs in high-impact journals and incorporation of TCM interventions into international clinical guidelines. These measures will help TCM achieve a paradigm shift from empirical medicine to evidence-based medicine, providing efficient and low-risk integrated strategies for global insomnia management.

## Data Availability

The original contributions presented in the study are included in the article/supplementary material, further inquiries can be directed to the corresponding author.
